# Interaction between vertebral artery hypoplasia and circle of Willis variants in posterior circulation stroke and TIA

**DOI:** 10.3389/fneur.2026.1752669

**Published:** 2026-02-11

**Authors:** Virginija Gaigalaite, Augenijus Vilimas, Jurate Dementaviciene, Sonata Varvuolyte, Inga Slautaite

**Affiliations:** 1 Institute of Clinical Medicine, Faculty of Medicine, Vilnius University, Vilnius, Lithuania; 2 Department of Radiology, Nuclear Medicine and Medical Physics, Faculty of Medicine, Vilnius University, Vilnius, Lithuania

**Keywords:** Circle of Willis, fetal-type circle of Willis, posterior circulation stroke, vertebral artery, vertebral artery hypoplasia

## Abstract

**Background:**

The clinical relevance of posterior Circle of Willis (CoW) variants remains controversial, particularly in the presence of vertebral artery hypoplasia (VAH). We aimed to evaluate whether posterior CoW configuration modifies the risk of posterior circulation stroke (PCS) or transient ischemic attack (TIA) depending on vertebral artery anatomy.

**Methods:**

In this retrospective case-control study, 1,339 participants were included, comprising 269 patients with PCS/TIA and 1,070 asymptomatic controls. All subjects underwent magnetic resonance or computed tomography angiography; extracranial vertebral arteries were additionally assessed by duplex ultrasonography. VAH was defined as a vertebral artery (VA) diameter < 2.5 mm along its entire course. Posterior CoW configuration was categorized as adult-type with both posterior communicating arteries (PComAs) present, adult-type with one or both PComAs absent, or fetal-type Circle of Willis. Multivariable logistic regression, stratified and interaction analyses were performed adjusting for age, sex, and classical vascular risk factors.

**Results:**

In the overall cohort, posterior CoW configuration was not independently associated with PCS/TIA after multivariable adjustment. Stratified analyses revealed marked effect modification by VAH. Among participants with VAH, adult-type configuration with both PComAs absent was associated with higher odds of PCS/TIA (OR 2.2, 95% CI 1.3–3.6), whereas fetal-type CoW was associated with a significantly lower risk (OR 0.35, 95% CI 0.17–0.70). In contrast, among participants without VAH, fetal-type CoW was associated with increased PCS/TIA risk (OR 1.76, 95% CI 1.04–2.9). Interaction analyses confirmed significant interactions between VAH and both absent PComAs and fetal-type CoW configurations.

**Conclusion:**

The clinical impact of posterior Circle of Willis variants is strongly context dependent. Fetal-type configuration appears protective in the presence of vertebral artery hypoplasia but may increase ischemic vulnerability in individuals with normal vertebral arteries, highlighting the importance of integrating vertebral artery anatomy when interpreting posterior Circle of Willis variants.

## 1 Introduction

Vertebral artery hypoplasia (VAH) is a relatively common anatomical variant of the vertebral arteries (VAs), characterized by reduced arterial diameter and asymmetrical vertebrobasilar flow. A recent meta-analysis demonstrated an association between VAH and posterior circulation infarction, suggesting that reduced vertebrobasilar inflow may contribute to ischemic vulnerability in the posterior circulation (1). Our previous work further showed a positive correlation between the degree of VA hypoplasia and the occurrence of posterior circulation stroke (PCS) or transient ischemic attack (TIA) (2).

Collateral circulation through the Circle of Willis (CoW) represents a key compensatory mechanism for maintaining cerebral perfusion in the setting of impaired inflow through major cerebral arteries. However, the functional and clinical relevance of posterior CoW variants—particularly in individuals with VAH—remains incompletely understood. Although the CoW is generally regarded as protective against ischemia, previous studies have reported inconsistent associations between posterior CoW anatomy and stroke risk, especially in the presence of vertebral artery hypoplasia (3–6).

The fetal-type configuration of the posterior CoW, in which the posterior cerebral artery originates predominantly from the internal carotid artery, represents a distinct collateral pattern that may substantially influence posterior circulation hemodynamics. Several studies have suggested that the coexistence of VAH and fetal-type CoW may predispose individuals to posterior circulation ischemia (4, 5), whereas others have reported no such association (6). Conversely, some investigations have proposed a protective role of fetal-type CoW, particularly under conditions of compromised vertebrobasilar perfusion (7). These conflicting observations underscore the complex interaction between vertebral artery anatomy and posterior CoW configuration in determining ischemic vulnerability.

Therefore, the aim of this retrospective study was to evaluate whether specific posterior Circle of Willis configurations modify the risk of posterior circulation stroke or transient ischemic attack depending on the presence or absence of vertebral artery hypoplasia. Given the inconsistent findings in previous studies, we hypothesized that the clinical relevance of posterior Circle of Willis variants is not uniform but depends on vertebral artery anatomy and posterior circulation inflow conditions. In addition, we sought to compare posterior Circle of Willis variants between individuals with and without vertebral artery hypoplasia to better understand how the compensatory capacity of the Circle of Willis varies according to vertebral artery anatomy.

**Figure 1:**
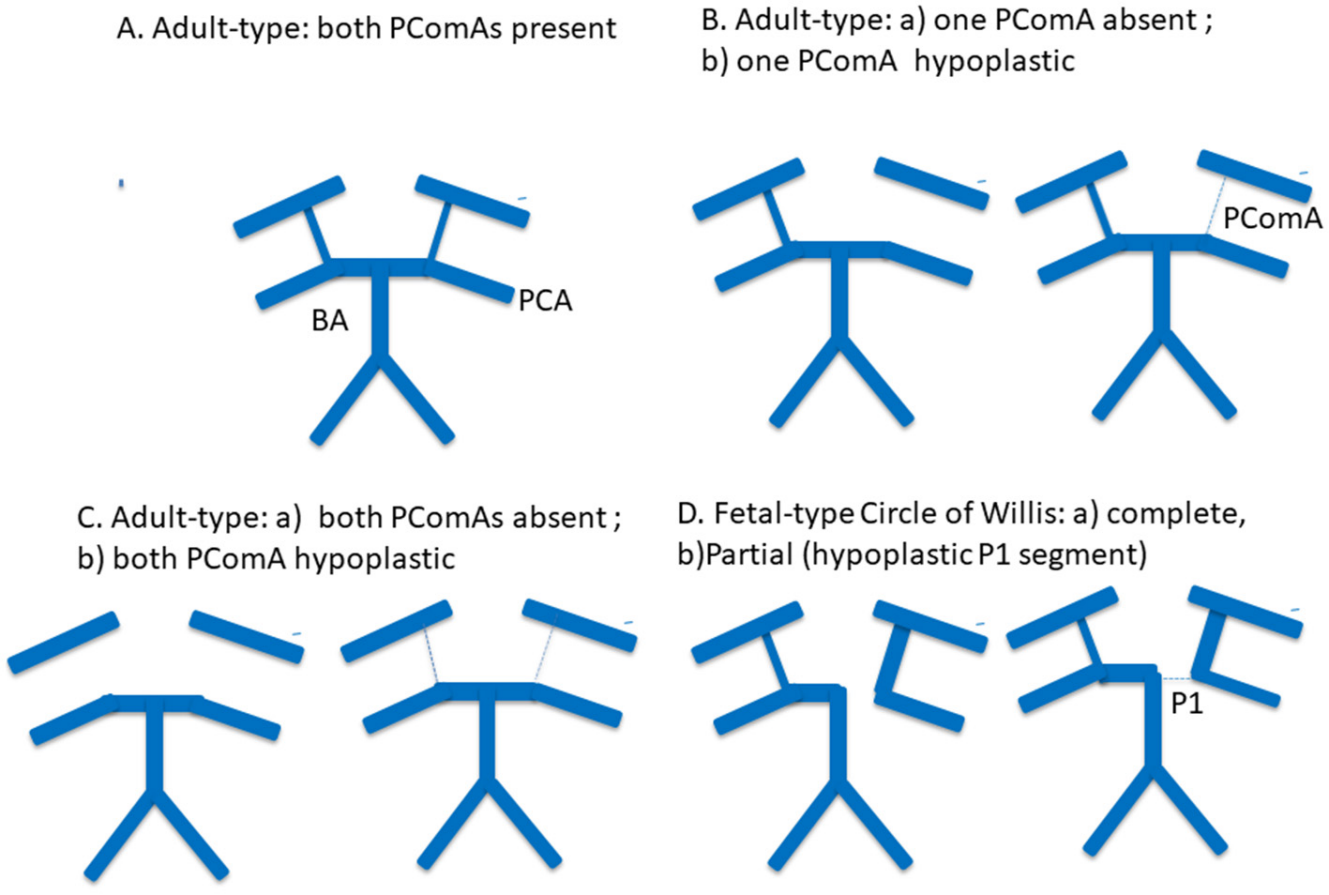
Schematic representation of posterior Circle of Willis configurations included in the analysis. Representative posterior Circle of Willis variants are shown, including a complete adult-type configuration, absence or hypoplasia of one posterior communicating artery (PComA), absence or hypoplasia of both PComAs, and fetal-type Circle of Willis configuration. Solid thick lines indicate normally developed arterial segments, thin or dashed lines indicate hypoplastic segments, and absent segments represent arterial aplasia. PComA, posterior communicating artery; P1, P1 segment of the posterior cerebral artery; PCA, posterior cerebral artery; BA, basilar artery.

Representative posterior Circle of Willis configurations included in the analysis are illustrated in Figure 1.

## 2 Materials and methods

### 2.1 Study design and participants

This retrospective case–control study included 1,339 participants who underwent brain MRI or CT with vascular imaging (MRA and/or CTA). The study population comprised 269 symptomatic patients with PCS or TIA and 1,070 asymptomatic controls.

Symptomatic patients were treated at Republican Vilnius University Hospital or Vilnius Clinical Hospital between 2021 and 2023. All symptomatic patients underwent CTA, and approximately half additionally underwent MRA.

Controls were identified from the neuroradiology picture archiving and communication system and consisted of inpatient and outpatient individuals who underwent brain MRA or CTA for non-cerebrovascular indications (e.g., headache, vertigo, and minor trauma). Control participants had no history of ischemic stroke or TIA in either anterior or posterior circulation and no significant (>50%) intracranial or extracranial arterial stenosis. In the control group, CTA was performed in 890 individuals (83.2%) and MRA in 425 individuals (39.7%).

Although control participants could have vascular comorbidities, this selection strategy was chosen to improve comparability with the symptomatic cohort regarding age distribution and vascular risk burden. Similar approaches have been used in prior cerebrovascular imaging studies (8). To mitigate potential selection bias, classical vascular risk factors were adjusted for in all multivariable models, and multiple prespecified sensitivity analyses were performed. This approach was chosen to balance anatomical comparability and vascular risk burden between groups, which is particularly important when evaluating anatomical variants rather than causal effects of vascular risk factors.

Participants were excluded if they:

1. did not undergo both brain imaging and vascular imaging;
2. had intracerebral hemorrhage or ischemic events confined to the anterior circulation;
3. had >50% stenosis or occlusion of the carotid, middle cerebral, or anterior cerebral arteries.

Stroke subtypes were classified according to TOAST criteria. Demographic and clinical data collected included age, sex, hypertension, diabetes mellitus, hypercholesterolemia, coronary artery disease, peripheral arterial disease, and atrial fibrillation.

### 2.2 Imaging protocol

#### 2.2.1 Magnetic resonance imaging and angiography

Brain MRI and MRA were performed using 1.5-T scanners (Philips Ingenia Ambition X or GE Optima MR450w). The protocol included T1-weighted, T2-weighted, FLAIR, diffusion-weighted imaging, susceptibility-weighted angiography, and 3D time-of-flight MRA. CoW anatomy was evaluated using both maximum intensity projection reconstructions and source images.

#### 2.2.2 Computed tomography angiography

CT and CTA were performed using a multidetector CT scanner (Toshiba Aquilion) following intravenous administration of non-ionic contrast. Image reconstruction included maximum intensity projection and volume-rendering techniques.

#### 2.2.3 Ultrasound examination

Duplex ultrasonography of extracranial carotid and vertebral arteries was performed using a Philips Affinity 70G system. Vertebral artery diameter measurements followed previously published methodology (2).

### 2.3 Image evaluation and anatomical classification

All MRA and CTA studies were independently reviewed by two experienced neuroradiologists blinded to clinical status. Discrepancies were resolved by consensus.

Posterior CoW configuration was classified according to established criteria (9, 10) and our prior work (11). Configurations included:

1. adult-type CoW with PComAs present;
2. adult-type CoW with one or both PComAs absent or hypoplastic;
3. fetal-type CoW.

Absent and hypoplastic PComAs were analyzed together, as both provide minimal collateral flow potential and may be inconsistently visualized across imaging modalities. Fetal-type CoW was defined as a posterior cerebral artery originating predominantly from the internal carotid artery, regardless of whether the P1 segment was absent or smaller than the PComA. Complete and partial fetal-type variants were analyzed as a single category.

VAH was assessed using MRA or CTA for the V4 segment and duplex ultrasonography for V1–V3 segments, applying previously validated criteria. Atherosclerotic involvement of the distal vertebral and basilar arteries was classified as normal (<50% stenosis), stenosis (≥50%), or occlusion.

### 2.4 Statistical analysis

Categorical variables were compared using χ^2^ or Fisher's exact tests, as appropriate. Continuous variables were assessed for normality and compared using independent-samples *t*-tests or Mann–Whitney *U*-tests. Data are presented as mean ± SEM or as counts and percentages.

Covariates were selected *a priori* based on clinical relevance and prior literature. All multivariable models included age and sex and were adjusted for hypertension, diabetes mellitus, coronary artery disease, hypercholesterolemia, and smoking. Peripheral arterial disease was not included due to low prevalence.

Atrial fibrillation and intracranial vertebrobasilar stenosis were treated as competing stroke mechanisms and were included in fully adjusted and sensitivity analyses but were not uniformly included in stratified or interaction models to avoid over adjustment and to facilitate clinical interpretability of effect modification.

Posterior CoW configuration was modeled as a categorical variable. Adult-type configurations were represented as a three-level variable based on PComA status, with fetal-type CoW modeled separately. Rare variants were excluded from regression analyses to avoid unstable estimates.

Primary multivariable logistic regression assessed associations between posterior CoW configuration and PCS/TIA in the overall cohort. Effect modification by VAH was evaluated using stratified analyses and multiplicative interaction terms. Different comparator definitions were prespecified to balance anatomical interpretability and statistical stability across analyses. Interaction significance was assessed using likelihood ratio testing.

Results are reported as ORs with 95% CIs. A two-sided *p* value < 0.05 was considered statistically significant. Analyses were performed using IBM SPSS Statistics version 24.

## 3 Results

### 3.1 Study population

A total of 1,339 participants were included in the analysis, comprising 269 symptomatic patients with posterior circulation ischemic stroke or transient ischemic attack (PCS/TIA) and 1,070 asymptomatic controls. VAH was present in 361 participants (27%). A complete posterior CoW configuration, defined by the presence of both PComAs, was observed in 209 individuals (15.6%). Baseline demographic and clinical characteristics stratified by symptomatic status and VAH status are presented in Table 1.

**Table 1:** Baseline characteristics of study participants stratified by symptomatic status and vertebral artery hypoplasia.

**Variable**	**Asymptomatic group (*n* = 1,070)**	**Symptomatic group (PCS/TIA) (*n* = 269)**	***p* value**	**VAH (*n* = 361)**	**Without VAH (*n* = 978)**	***p* value**
Age (years), mean ± SEM	55.4 ± 0.3	63.5 ± 0.7	0.001	56.9 ± 0.6	57.5 ± 0.3	0.3
Gender, female sex (%)	52.7	47.6	0.13	57.6	49.5	0.008
Arterial hypertension (%)	55.0	69.9	0.001	56.8	58.5	0.57
Diabetes (%)	10.6	14.1	0.098	10.0	11.8	0.36
Coronary artery disease (%)	15.1	19.7	0.062	14.4	16.6	0.34
Hypercholesterolemia (%)	47.1	52.4	0.12	45	49.3	0.16
Peripheral vascular disease (%)	0.75	1.9	0.15	1.1	0.92	0.7
Smoking (%)	25	27.1	0.46	22.4	26.5	0.13
Atrial fibrillation (%)	8.8	13	0.036	7.5	10.4	0.1

VAH, vertebral artery hypoplasia; PCS, posterior circulation stroke; TIA, transient ischemic attack; SEM, standard error of the mean.

Participants with VAH were more frequently female compared with those without VAH (57.6 vs. 49.5%, *p* = 0.008). Symptomatic patients were older and more frequently had arterial hypertension and atrial fibrillation than asymptomatic controls. VAH was predominantly unilateral; bilateral hypoplasia was rare overall but occurred more frequently among symptomatic participants than asymptomatic controls (5.9 vs. 1.2%, *p* = 0.01). Given the very limited number of cases, bilateral hypoplasia was not modeled as a separate exposure.

Among symptomatic participants, 109 (40.5%) were diagnosed with posterior circulation stroke and 160 (59.5%) with transient ischemic attack. Stroke etiology was classified as large-vessel disease in 44.4%, cryptogenic in 35.8%, small-vessel disease in 11.9%, and cardioembolic in 8.3% of cases.

### 3.2 Posterior Circle of Willis variants and symptomatic status

The distribution of posterior CoW configurations differed modestly between symptomatic and asymptomatic participants (Table 2). Absence of both PComAs was more frequent among symptomatic patients compared with controls (54.6 vs. 46.7%, *p* = 0.02). In contrast, the prevalence of fetal-type Circle of Willis did not differ significantly between groups (15.6 vs. 16.6%, *p* = 0.68).

**Table 2:** Distribution of posterior Circle of Willis configurations by symptomatic status.

**Configuration of posterior part of Circle of Willis**	**Asymptomatic group (*n* = 1070)**	**Symptomatic group (PCS/TIA) (*n* = 269)**	***p* value**
Adult-type, both PComAs present, *n* (%)	169 (15.8)	37 (13.8)	0.40
Adult-type, one PComA absent, *n* (%)	223 (20.8)	43 (16)	0.08
Adult-type, both PComAs absent, *n* (%)	500 (46.7)	147 (54.6)	0.02
Fetal-type CoW, *n* (%)	178 (16.6)	42 (15.6)	0.68
Fetal-type CoW + A1 aplasia of ACA, *n* (%)	8 (0.75)	7 (2.6)	0.02

CoW, circle of Willis; PComA, posterior communicating artery; ACA, anterior cerebral artery; PCS, posterior circulation stroke; TIA, transient ischemic attack.

A rare configuration consisting of fetal-type CoW combined with aplasia of the A1 segment of the anterior cerebral artery was observed more frequently among symptomatic participants (2.6 vs. 0.75%, *p* = 0.02). Due to the small number of cases, this variant was excluded from regression analyses.

### 3.3 Multivariable association between posterior Circle of Willis configuration and PCS/TIA

In multivariable logistic regression analyses adjusted for age, sex, and classical vascular risk factors, posterior Circle of Willis configuration was not significantly associated with symptomatic posterior circulation stroke or transient ischemic attack in the overall cohort (Table 3). Compared with participants with adult-type configuration and both PComAs present, neither adult-type configurations with one or both PComAs absent nor fetal-type Circle of Willis configuration demonstrated a statistically significant association with symptomatic status.

**Table 3:** Association between posterior Circle of Willis configuration and posterior circulation stroke/TIA in multivariable models.

**Configuration of posterior part of Circle of Willis**	**Model 1**	***p* value**	**Model 2**	***p* value**
	**Adjusted OR (95% CI)**		**Fully adjusted OR (95% CI)**	
Adult-type, both PComAs present	Reference	**-**	Reference	
Adult-type, one PComA absent	0.9 (0.6–1.6)	0.8	0.8 (0.4–1.9)	0.6
Adult-type, both PComAs absent	1.4 (0.8–2.2)	0.2	1.2 (0.5–2.5)	0.5
Fetal-type CoW	1.3 (0.8–2.3)	0.3	1.03 (0.5–3.3)	0.8

Results are presented as odds ratios (ORs) with 95% confidence intervals (CIs). Posterior communicating artery status in adult-type Circle of Willis was modeled as a three-level categorical variable [both PComAs present (reference), one PComA absent, both PComAs absent]. Fetal-type Circle of Willis configuration was analyzed as a separate category.

Model 1 was adjusted for age, sex, arterial hypertension, diabetes mellitus, coronary artery disease, hypercholesterolemia, and smoking. Model 2 was additionally adjusted for atrial fibrillation and intracranial vertebrobasilar stenosis as competing stroke mechanisms.

Additional adjustment for atrial fibrillation and intracranial vertebrobasilar stenosis yielded similar effect directions with wider confidence intervals (Table 3), consistent with attenuation of Circle of Willis–related associations after accounting for competing stroke mechanisms.

Given the strong association of atrial fibrillation and intracranial vertebrobasilar stenosis with posterior circulation ischemic events, these variables were treated as competing stroke mechanisms and were further explored in prespecified sensitivity analyses.

Prespecified sensitivity analyses using alternative comparator definitions demonstrated that, when adult-type configuration with both PComAs absent was compared against all other Circle of Willis configurations combined, the association approached statistical significance in the overall cohort (OR 1.38, 95% CI 0.99–1.80; *p* = 0.054; Supplementary Table S1). This finding should therefore be interpreted cautiously.

In analyses excluding participants with atrial fibrillation, adult-type configuration with both PComAs absent showed a stronger association with symptomatic posterior circulation events. While the primary contrast using a single reference group did not reach statistical significance (OR 1.6, 95% CI 0.92–3.0; Supplementary Table S2), the sensitivity contrast comparing this configuration against all other configurations combined was statistically significant (OR 1.46, 95% CI 1.03–2.0; Supplementary Table S2).

Fully adjusted models confirmed atrial fibrillation and intracranial vertebrobasilar stenosis as strong independent correlates of posterior circulation ischemic events, whereas effect estimates for posterior Circle of Willis configurations were further attenuated (Supplementary Table S3). In sensitivity analyses restricted to participants without intracranial vertebrobasilar stenosis, no significant associations between posterior Circle of Willis configuration and PCS/TIA were observed using either primary or alternative contrast definitions (Supplementary Table S4).

### 3.4 Stratified analyses according to vertebral artery hypoplasia

Stratified analyses demonstrated clear heterogeneity in associations between posterior CoW configuration and PCS/TIA according to VAH status (Table 4). Table 4 presents sensitivity contrasts comparing each configuration against all other configurations combined to improve estimate stability within strata. Among participants with VAH, adult-type configuration with both PComAs absent was associated with higher odds of PCS/TIA (OR 2.2, 95% CI 1.3–3.6), whereas fetal-type CoW was associated with lower odds (OR 0.35, 95% CI 0.17–0.70). Among participants without VAH, adult-type configuration with both PComAs absent was not associated with PCS/TIA (OR 1.02, 95% CI 0.65–2.2), while adult-type configuration with one PComA absent showed borderline lower odds (OR 0.6, 95% CI 0.3–0.9; *p* = 0.05). In contrast, fetal-type CoW was associated with increased odds of PCS/TIA (OR 1.76, 95% CI 1.04–2.9). Reference-based stratified models using adult-type configuration with both PComAs present as the reference category (anatomically interpretable contrasts) and fully adjusted stratified models including atrial fibrillation and vertebrobasilar stenosis are provided in Supplementary Tables S5, S6; effect directions were consistent with attenuation after adjustment for competing mechanisms. Consistent effect directions across reference-based and sensitivity stratified models support the robustness of these findings.

**Table 4:** Stratified association between posterior Circle of Willis configuration and PCS/TIA by vertebral artery hypoplasia.

**Configuration of posterior part of Circle of Willis**	**VAH: OR (95% CI)**	***P* value**	**Without VAH: OR (95% CI)**	***P* value**
Adult-type, one PComA absent	0.9 (0.5–1.8)	0.7	0.6 (0.3–0.9)	0.05
Adult-type, both PComAs absent	2.2 (1.3–3.6)	0.004	1.02 (0.65–2.2)	0.8
Fetal-type CoW	0.35 (0.17–0.7)	0.004	1.76 (1.04–2.9)	0.034

Stratified multivariable logistic regression models comparing each posterior Circle of Willis configuration with all other configurations combined within each vertebral artery hypoplasia stratum. Models were adjusted for age, sex, arterial hypertension, diabetes mellitus, coronary artery disease, hypercholesterolemia, and smoking.

PComA, posterior communicating artery; VAH, vertebral artery hypoplasia; PCS, posterior circulation stroke; TIA, transient ischemic attack.

### 3.5 Interaction analysis

Formal interaction testing confirmed effect modification by VAH (Table 5). Statistically significant interactions were observed for adult-type CoW with both PComAs absent and for fetal-type CoW, indicating that associations differed by VAH status. The primary interaction models used contrasts comparing each configuration against all other configurations combined to optimize estimate stability. Sensitivity interaction models using a single reference group (adult-type with both PComAs present) are shown in Supplementary Table S7, with consistent direction of interaction effects. Alternative comparator definitions were used in interaction models to optimize statistical power without altering the direction of observed effects.

**Table 5:** Interaction between vertebral artery hypoplasia and posterior Circle of Willis configuration in relation to posterior circulation stroke/TIA.

**Configuration of posterior part of Circle of Willis**	**Interaction OR (VAH × CoW) (95% CI)**	***p* for interaction**
Adult-type, one PComA absent	1.9 (0.7–5.3)	0.2
Adult-type, both PComAs absent	2.2 (1.1–3.5)	0.03
Fetal-type CoW	0.2 (0.1–0.46)	0.001

Interaction terms were obtained from multivariable logistic regression models including an interaction term between vertebral artery hypoplasia and the corresponding posterior Circle of Willis configuration. Models were adjusted for age, sex, arterial hypertension, diabetes mellitus, coronary artery disease, hypercholesterolemia, and smoking. For interaction testing, Circle of Willis configurations were compared against all other configurations combined to improve estimate stability and statistical power.

**Figure 2:**
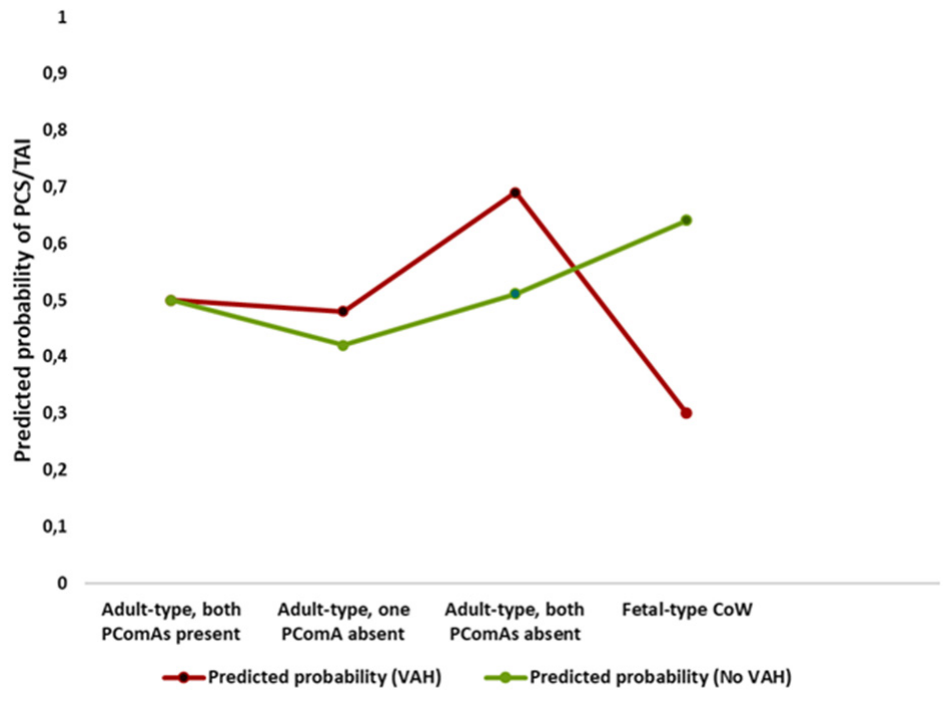
Interaction between posterior Circle of Willis configuration and vertebral artery hypoplasia on predicted probability of posterior circulation stroke or transient ischemic attack. Predicted probabilities of posterior circulation stroke or transient ischemic attack across posterior Circle of Willis configurations, stratified by vertebral artery hypoplasia (VAH), demonstrating opposite risk patterns for fetal-type Circle of Willis in participants with and without VAH.

These opposing associations are illustrated in Figure 2, which shows predicted probabilities of posterior circulation stroke/TIA across posterior Circle of Willis configurations stratified by VAH.

## 4 Discussion

In this study, we demonstrate that anatomical variants of the posterior Circle of Willis (CoW) are highly prevalent in both asymptomatic individuals and patients with posterior circulation stroke or transient ischemic attack (PCS/TIA). More than four-fifths of participants exhibited some degree of posterior CoW variation, confirming that incomplete posterior configurations represent a common anatomical pattern rather than an exception. Importantly, our findings indicate that the clinical relevance of these variants is strongly context dependent and critically influenced by vertebral artery anatomy and competing vascular mechanisms.

### 4.1 Posterior Circle of Willis incompleteness and PCS/TIA association

A completely incomplete posterior CoW, defined by the absence of both PComAs, was more frequently observed among symptomatic individuals than asymptomatic controls. This observation is consistent with previous reports and meta-analytic evidence suggesting a modest association between posterior CoW incompleteness and ischemic stroke (3), as well as studies demonstrating a higher prevalence of CoW variants among stroke patients (8).

However, after adjustment for age, sex, and classical vascular risk factors, posterior CoW incompleteness was not independently associated with PCS/TIA in the overall cohort. Sensitivity analyses using alternative comparator definitions yielded slightly stronger but still modest associations, underscoring that posterior CoW anatomy alone is insufficient to substantially increase ischemic risk in an unselected population.

Notably, associations between posterior CoW configuration and posterior circulation ischemic events were more apparent in analyses excluding atrial fibrillation. This finding suggests that posterior CoW variants may be more relevant for non-cardioembolic, hemodynamically mediated ischemic events, rather than serving as independent risk factors across all stroke mechanisms.

### 4.2 Context-dependent role of fetal-type Circle of Willis

In the overall cohort, fetal-type CoW configuration was not independently associated with PCS/TIA, supporting the notion that posterior CoW variants do not exert uniform effects across different anatomical and clinical contexts. This lack of a main effect contrasts with the pronounced heterogeneity observed after stratification by vertebral artery hypoplasia (VAH), highlighting the importance of vertebral artery anatomy in modulating the functional relevance of CoW configurations.

### 4.3 Effect modification by vertebral artery hypoplasia

Stratified and interaction analyses consistently demonstrated that vertebral artery hypoplasia (VAH) appears to function as an effect modifier in the association between posterior Circle of Willis configuration and posterior circulation stroke or TIA. Among individuals with VAH, an adult-type configuration with absence of both posterior communicating arteries (PComAs) was associated with higher odds of symptomatic posterior circulation events in our cohort, whereas a fetal-type Circle of Willis configuration was associated with was associated with a significantly lower likelihood of PCS/TIA in this subgroup. In contrast, among individuals without VAH, adult-type configurations with absence of both PComAs were not associated with higher odds of PCS/TIA, while a fetal-type Circle of Willis configuration was associated with increased odds of symptomatic posterior circulation events.

These contrasting patterns were confirmed by formal interaction testing and remained directionally consistent across multiple sensitivity analyses, supporting the robustness of the observed effect modification. From a hemodynamic perspective, this pattern suggests that PComAs may assume a compensatory role primarily in the presence of an underdeveloped vertebrobasilar system, whereas in individuals with normal vertebral artery diameters, the absence of one or both PComAs appears to have limited hemodynamic relevance.

### 4.4 Hemodynamic interpretation

The divergent effects of posterior CoW configurations across VAH strata likely reflect differences in posterior circulation inflow and collateral demands. In individuals with VAH, vertebrobasilar inflow is reduced, increasing reliance on collateral pathways. In this setting, fetal-type CoW may confer a compensatory advantage by enabling dominant posterior cerebral artery perfusion from the internal carotid system via enlarged PComAs, thereby preserving posterior circulation flow despite compromised vertebral artery input.

This interpretation is supported by our previous findings demonstrating an increasing prevalence of fetal-type CoW with more pronounced vertebral artery hypoplasia among asymptomatic individuals (11), as well as by studies suggesting that persistent fetal-type circulation may enhance collateral connectivity when posterior inflow is limited (7). Conversely, in individuals with normal vertebral artery diameters, vertebrobasilar flow is typically sufficient under resting conditions. In such cases, fetal-type CoW may limit adaptive collateral recruitment during posterior circulation compromise by functionally segregating anterior and posterior circulatory territories, rendering individuals more susceptible to posterior circulation ischemia.

Importantly, these findings do not contradict reports linking combined hypoplasia of the vertebrobasilar system and fetal-type CoW with posterior circulation ischemia (5). Rather, they emphasize that the hemodynamic consequences of fetal-type CoW are context specific and depend on the overall balance between posterior inflow and collateral demand.

## 5 Clinical implications

Taken together, these findings indicate that posterior Circle of Willis variants should not be interpreted as static anatomical risk factors. Instead, their clinical relevance depends on vertebral artery anatomy and the presence or absence of competing stroke mechanisms. Incorporating vertebral artery morphology into the interpretation of posterior CoW configurations may improve risk stratification and provide a more nuanced understanding of posterior circulation ischemia.

## 6 Limitations and future directions

Several limitations should be acknowledged. Vessel diameter measurements derived from CTA and MRA may be subject to technical variability; however, grouping absent and hypoplastic segments and analyzing fetal-type configurations as unified anatomical categories likely reduced modality-related bias (8). The study did not include functional hemodynamic measurements, which could clarify the physiological significance of anatomical variants. In addition, the retrospective case–control design limits causal inference. The relatively small number of participants with bilateral vertebral artery hypoplasia or severe intracranial vertebrobasilar stenosis may further limit generalizability to higher-risk populations.

Future prospective studies incorporating quantitative perfusion imaging and longitudinal follow-up are needed to determine whether specific posterior CoW configurations predict stroke risk, severity, or recovery, particularly in individuals with coexisting vertebral artery hypoplasia and intracranial or extracranial stenosis.

## 7 Conclusion

The compensatory capacity of posterior Circle of Willis variants is not fixed but depends on vertebral artery anatomy and competing vascular mechanisms. Adult-type posterior CoW incompleteness alone appears to be associated with only modest risk, whereas fetal-type Circle of Willis configuration may be protective in the presence of vertebral artery hypoplasia and disadvantageous in individuals with normal vertebral arteries. These findings highlight the importance of integrating vertebral artery anatomy when interpreting posterior Circle of Willis variants in posterior circulation ischemia.

## Data Availability

The raw data supporting the conclusions of this article will be made available by the authors, without undue reservation.
