# Supplementary material for: Interaction between vertebral artery hypoplasia and circle of Willis variants in posterior circulation stroke and TIA
Source: Front Neurol. 2026 Feb 11;17:1752669. doi: 10.3389/fneur.2026.1752669 (PMC12933647; doi:10.3389/fneur.2026.1752669)
Supplement: Supplementary file 1 [file Data_Sheet_1.pdf]

Supplementary analyses

Supplementary analyses were prespecified to evaluate the robustness of the primary findings and to facilitate interpretation of potentially context-dependent effects**.** Alternative contrast definitions of posterior Circle of Willis (CoW) configuration were applied, including reference-based models (adult-type configuration with both posterior communicating arteries (PComAs) present) and sensitivity contrasts comparing each configuration against all other configurations combined to improve estimate stability. Additional sensitivity analyses excluded participants with atrial fibrillation and restricted analyses to individuals without intracranial vertebrobasilar stenosis to account for competing stroke mechanisms.

Stratified and interaction analyses according to vertebral artery hypoplasia (VAH) were conducted using prespecified multivariable models adjusted for age, sex, and classical vascular risk factors. Fully adjusted stratified and interaction models additionally including atrial fibrillation and intracranial vertebrobasilar stenosis were performed as sensitivity analyses to assess the robustness of the observed effect modification.

Supplementary Table S1 presents prespecified alternative contrasts of posterior CoW configuration to assess the robustness of primary findings to comparator definition. Primary contrasts used the adult-type configuration with both PComAs present as the reference (complete posterior collateral anatomy). Because this subgroup was relatively small, sensitivity contrasts compared each configuration against all other configurations combined to improve estimate stability.

Supplementary Table S1. **Alternative contrasts of posterior Circle of Willis configuration and posterior circulation stroke or TIA (PCS/TIA)**

S1A. Primary contrasts (single reference group)

**Posterior CoW category (4-level)**	**Reference**	**Adjusted OR (95% CI)**	**p value**
Adult-type, one PComA absent	Adult-type, both PComAs present	0.9 (0.6–1.6)	0.8
Adult-type, both PComAs absent	Adult-type, both PComAs present	1.4 (0.8–2.2)	0.2
Fetal-type CoW	Adult-type, both PComAs present	1.3 (0.8-2.3)	0.3

S1B Sensitivity contrasts (each category vs all other configurations combined)

**Contrast (binary exposure)**	**Comparator**	**Adjusted OR (95% CI)**	**p value**

Adult-type, one PComA absent	All other configurations	0.7 (0.5–1.04)	0.1
Adult-type, both PComAs absent	All other configurations	1.38 (0.99-1.8)	0.054
Fetal-type CoW	All adult-type configurations	0.96 (0.62-1.5)	0.88

Footnote (S1): Models were adjusted for age, sex, arterial hypertension, diabetes mellitus, coronary artery disease, hypercholesterolemia, and smoking.

Supplementary Table S2 repeats the primary and sensitivity contrasts after excluding participants with atrial fibrillation to reduce the influence of cardioembolic stroke mechanisms.

.

Supplementary Table S2. Association between posterior Circle of Willis configuration and PCS/TIA after exclusion of atrial fibrillation

S2A. Primary contrasts (single reference group)

**Posterior CoW category**	**Adjusted OR (95% CI)**	**p value**
Adult-type, both PComAs present	Reference	
Adult-type, one PComA absent	1.1 (0.5-2.2)	0.7
Adult-type, both PComAs absent	1.6 (0.92-3.0)	0.09
Fetal-type CoW	1.46 (0.8-2.8)	0.2

S2B. Sensitivity contrasts (vs all other configurations)

**Posterior CoW category**	**Adjusted OR (95% CI)**	**p value**
Adult-type, one PComA absent	0.9 (0.6-1.1)	0.5

Adult-type, both PComAs absent	1.46 (1.03-2)	0.035
Fetal-type CoW	0.9 (0.7-1.5)	0.8

Footnote (S2): Models were adjusted for age, sex, arterial hypertension, diabetes mellitus, coronary artery disease, hypercholesterolemia, and smoking. Participants with atrial fibrillation were excluded.

Supplementary Table S3 presents a fully adjusted multivariable logistic regression model including atrial fibrillation and intracranial vertebrobasilar stenosis as competing stroke mechanisms. These variables demonstrated strong independent associations with posterior circulation ischemic events and attenuated the associations between posterior CoW configuration and PCS/TIA.

Supplementary Table S3. Fully adjusted model including atrial fibrillation and intracranial vertebrobasilar stenosis

**Variable**	**Adjusted OR (95% CI)**	**p value**
Age, per year	1.03 (1.01-1.06)	0.001
Sex (female)	0.54 (0.32-0.9)	0.022
Vertebrobasilar stenosis	3.9 (1.3-12)	0.002
Atrial fibrillation	3.9 (1.7-8.9)	0.021
Adult-type, both PComAs absent *	1.35 (0.8-2.3)	0.26

Footnote (S3*):* Fully adjusted multivariable logistic regression including atrial fibrillation and intracranial vertebrobasilar stenosis. A representative CoW contrast is shown; other CoW contrasts demonstrated similar attenuation.

Supplementary Table S4 presents analyses restricted to participants without intracranial vertebrobasilar stenosis to evaluate CoW associations in a hemodynamically less confounded subgroup (adjusted for age, sex, and classical vascular risk factors)..

Supplementary Table S4. Association between posterior Circle of Willis configuration and PCS/TIA in participants without intracranial vertebrobasilar stenosis

S4A. Primary contrasts (single reference group)

**Posterior CoW category**	**Adjusted OR (95% CI)**	**p value**
Adult-type, both PComAs present	Reference	
Adult-type, one PComA absent	0.8 (0.3-2.1)	0.65
Adult-type, both PComAs absent	1.2 (0.7-2.2)	0.4
Fetal-type CoW	0.9 (0.4-2.5)	0.9

S4B. Sensitivity contrasts (vs all other configurations)

**Posterior CoW category**	**Adjusted OR (95% CI)**	**p value**
Adult-type, one PComA absent	0.7 (0.3-1.4)	0.3
Adult-type, both PComAs absent	1.4 (0.98-2.1)	0.06
Fetal-type CoW	0.9 (0.4-1.4)	0.5

Footnote (S4): Analyses were restricted to participants without intracranial vertebrobasilar stenosis and adjusted for age, sex, and classical vascular risk factors. Atrial fibrillation was not included, as its influence was examined separately.

Supplementary Table S5 provides stratified analyses by vertebral artery hypoplasia (VAH) using a single reference group (adult-type with both PComAs present) as a sensitivity analysis complementing the main stratified results presented in Table 4.

Supplementary Table S5. Stratified multivariable models by vertebral artery hypoplasia using a single reference group (adult-type configuration with both PComAs present)

Posterior CoW category	VAH, OR (95% CI)		P value	Without VAH, OR (95% CI)	p value
Adult-type, both PComAs present	Reference			Reference	
Adult-type, one PComA absent	1.2 (0.5-3.7)		0.5	0.7 (0.3-1.6)	0.3
Adult-type, both PComAs absent	1.8 (0.8-4.1)		0.15	1.02 (0.5-2.1)	0.92
Fetal-type CoW	0.4 (0.2-0.9)		0.03	2.1(1.0-5.0)	0.041

Footnote (S5): Models were adjusted for age, sex, arterial hypertension, diabetes mellitus, coronary artery disease, hypercholesterolemia, and smoking. Adult-type configuration with both PComAs present served as the reference group within each VAH stratum.

Supplementary Table S6 provides fully adjusted stratified models by VAH including atrial fibrillation and vertebrobasilar stenosis to assess robustness to competing mechanisms.

Supplementary Table S6. Fully adjusted stratified multivariable models by vertebral artery hypoplasia including atrial fibrillation and intracranial vertebrobasilar stenosis.

S6A. Reference-based contrasts (adult-type configuration with both PComAs present)

Posterior CoW category	VAH: Adjusted OR (95% CI)	p value	Without VAH: Adjusted OR (95% CI)	p value
Adult-type, both PComAs present	Reference		Reference	
Adult-type, one PComA absent	1.5 (0.4-10)	0.6	0.7 (0.1-3)	0.5
Adult-type, both PComAs absent	1.9 (0.8-4.7)	0.15	0.9 (0.3-3.4)	0.8
Fetal-type CoW	0.5 (0.1-1.8)	0.3	1.7 (0.7-4.1)	0.2

S6B. Sensitivity contrasts (vs all other configurations)

Posterior CoW category	VAH: Adjusted OR (95% CI)	p value	Without VAH: Adjusted OR (95% CI)	p value
Adult-type, one PComA absent	1.2 (0.2-5.1)	0.8	0.5 (0.2-1)	0.1
Adult-type, both PComAs absent	2.2 (1.3-3.9)	0.007	0.9 (0.4-2)	0.7
Fetal-type CoW	0.38 (0.18-0.8)	0.016	1.5 (0.8-2.7)	0.16

Footnote (S6): Stratified models additionally adjusted for atrial fibrillation and intracranial vertebrobasilar stenosis. Results are presented as odds ratios (ORs) with 95% confidence intervals (CIs).

Supplementary Table S7 presents interaction models using a single reference group (adult-type with both PComAs present), provided as a sensitivity analysis complementing the main interaction results shown in Table 5.

Supplementary Table S7. Interaction between vertebral artery hypoplasia and posterior Circle of Willis configuration using a single reference group.

Posterior CoW category	Interaction OR (VAH × CoW configuration) (95% CI)	p for interaction
Adult-type, one PComA absent	0.7 (0.3-1.5)	0.3
Adult-type, both PComAs absent	1.8 (0.8–4.8)	0.05
Fetal-type CoW	0.21 (0.08-0.7)	0.002

Footnote (S7): Interaction ORs were obtained from multivariable logistic regression models including VAH, posterior CoW configuration (adult-type with both PComAs present as the

reference), and VAH×CoW multiplicative interaction terms, adjusted for age, sex, arterial hypertension, diabetes mellitus, coronary artery disease, hypercholesterolemia, and smoking.
